# Survival of NSCLC Patients Treated with Cimavax-EGF as Switch Maintenance in the Real-World Scenario

**DOI:** 10.7150/jca.67189

**Published:** 2023-04-01

**Authors:** Yoanna I. Flores Vega, Diana L. Páramo González, Sofía C Alsina Sarmiento, Luis E. Alsina Tul, Iris B. Inguanzo Valdés, Jorge Rodríguez Machado, Ángel Elejalde Larrinaga, Justo E Flores Rodríguez, Janet Lamadrid García, Danay Corrales Otero, Ramón Ropero Toirac, Tania Crombet Ramos, Elias A. Gracia Medina

**Affiliations:** 1 Department of Medical Oncology. National Institute of Oncology and Radiobiology. 29th Street, corner E, Vedado Plaza de la Revolución, Havana Cuba; 2 Department of Radiotherapy. National Institute of Oncology and Radiobiology. 29th Street, corner E, Vedado Plaza de la Revolución, Havana Cuba; 3 Department of Pneumology. National Institute of Oncology and Radiobiology. 29th Street, corner E, Vedado Plaza de la Revolución, Havana Cuba.; 4 Department of Pathology, National Institute of Oncology and Radiobiology. 29th Street, corner E, Vedado Plaza de la Revolución, Havana Cuba.; 5 Department of Imaging, National Institute of Oncology and Radiobiology. 29th Street, corner E, Vedado Plaza de la Revolución, Havana Cuba.; 6 Department of Clinical Trials, National Institute of Oncology and Radiobiology. 29th Street, corner E, Vedado Plaza de la Revolución, Havana Cuba.; 7 Clinical Direction. Center of Molecular Immunology, Ave 216, Esq 15. Atabey. Playa

**Keywords:** CIMAvax-EGF, NSCLC, EGFR, vaccine, immunotherapy

## Abstract

**Introduction**: In Cuba, lung cancer represents the first cause of mortality for both sexes. Non-small cell lung cancer (NSCLC) is the most prevalent histology. Overall, 75-85% of NSCLC overexpress EGFR and its ligands. EGFR overexpression has been implicated in the malignant transformation by promoting cell proliferation and survival. CIMAvax-EGF is a therapeutic vaccine composed of recombinant-human EGF conjugated to a carrier protein and Montanide as an adjuvant. CIMAvax-EGF is intended to induce antibodies against self-EGF that block the EGF-EGFR interaction.

**Objectives:** To characterize the efficacy and safety of CIMAvax-EGF as maintenance in NSCLC patients treated in the real-world setting.

**Results:** 106 patients diagnosed with advanced NSCLC at the National Institute of Oncology and Radiobiology, who had at least stable disease after first-line therapy, were enrolled in the study. The initial four CIMAvax-EGF doses were administered every 2 weeks and then, patients received monthly re-immunizations. Globally, 52.8% of the patients were 65 years or older, 77.4% had an ECOG 1 and 62.3% had an adenocarcinoma. The median survival time (MST) was 14.6 months. Patients younger than 65 years had a MST of 16.7 months and subjects with ECOG 0 survived for 29 months. The median progression-free survival was 8.16 months. Overall, 36.8% and 19.8% of patients maintained disease control at 6 and 12 months, respectively. The most frequent adverse events were pain (27.3%) or induration (7.3%) at the injection site and local erythema (10.9%).

**Conclusion:** CIMAvax-EGF, as an EGF depleting immunotherapy used as switch-maintenance was safe and effective in patients with NSCLC.

## Introduction

Lung cancer continues to be a global health problem. In 2020, 2,206,771 (11.4%) new patients with lung cancer were diagnosed. In addition, 1,796,144 (18.4%) deaths were reported from this pathology. Only 18% of patients survive more than five years after diagnosis 1.

In Cuba, lung cancer represents the first cause death for cancer in both sexes. According the 2019 statistical yearbook, there were 5026 deaths from this disease, which represents an adjusted crude rate to the world population (ACR) of 50 x 100 000 inhabitants 2.

Regarding incidence, lung cancer represents the third cause, preceded by skin cancer for both sexes and prostate cancer for men and breast cancer for women. In 2016, 3735 new male cases were diagnosed, representing an ACR of 68.5 x 100 000 inhabitants. In the case of females, 2176 new cases were reported, for an ACR of 20.7 x 100 000 inhabitants 2.

Using maintenance therapies after platinum-based chemotherapy as first line, increased the survival of those patients showing stable disease or better 3 4. Pemetrexed and bevacizumab, as well as tyrosine kinase inhibitors have shown clinical benefit in these patients 5-7.

Approximately 75-85% of non-small cell lung cancer (NSCLC) patients overexpress the epidermal growth factor receptor (EGFR) and its ligands. This overexpression is involved in the malignant transformation process, by promoting cell proliferation and survival. EGFR is a well-validated therapeutic target in patients with NSCLC 8, 9.

Immunotherapy has played an important role in the treatment of lung cancer. The main benefit of specific active immunotherapy is the ability to direct the immune response to the individual's own tumor. The induction of EGF deprivation by active immunotherapy is an emerging concept developed by Cuban researchers, which includes the manipulation of the individual's immune response to generate neutralizing antibodies (Abs) against self-EGF that reduce the size of the tumor or prevent its progression 8, 9.

CIMAvax-EGF consists of a chemical conjugate between EGF and P64, a recombinant protein from Neisseria meningitides and an adjuvant (Montanide ISA51, VG). The vaccine induces antibodies against EGF, capable of blocking the EGF-EGFR interaction 10, 11.

From 1995, our group is participating in the clinical trials to assess the efficacy and safety of the drug 12-14. In 2008, the State Center for the Drug Quality Control and medical devices (CECMED), the Cuban regulatory agency, approved a conditional registration. In 2014, the definitive approval as maintenance treatment of NSCLC was granted by CECMED. In 2015, CIMAvax-EGF commercialization begins. The main objective of this study was to evaluate the efficacy and safety of the CIMAvax-EGF in patients with NSCLC in the real-world scenario.

## Patients and methods

An observational, descriptive, longitudinal study was conducted in patients with NSCLC in stages IIIB and IV at the National Institute of Oncology and Radiobiology, from January 2015 to December 2017. Patients older than 18 years diagnosed with NSCLC, in clinical stage IIIB and IV, who have had an objective response or at least stabilization of the disease to first-line treatment were enrolled. Patients with brain metastases or progressive disease at the end of first-line treatment were excluded.

A hundred and six (106) patients were included. They were treated with CIMAvax-EGF after showing an objective response or disease stabilization to first line therapy including platinum doublets, chemotherapy and radiotherapy or single agent chemotherapy.

The study was approved by the scientific council, as well as by the ethics committee of the institution. All included patients signed the informed consent for the investigation. The study was conducted according to the Helsinki ethical principles for medical research involving human subjects. The study was funded by the Cuban Ministry of Health.

Patients received CIMAvax-EGF after the administration of cyclophosphamide at a dose of 200 mg/m^2^ on day 0. Three days after, they started CIMAvax-EGF administration every 14 days for 4 doses and subsequently, every 28 days until disease progression or unacceptable toxicity.

The response to treatment was evaluated according to the Response Evaluation Criteria in Solid Tumors (version 1.1) (RECIST v1.1), at 6 months and 1 year after treatment.

### Statistical analysis

Categorical variables were displayed using descriptive summary statistics including number of patients and percentages while metric variables were presented using arithmetic mean ± standard deviation. Overall survival and progression-free survival were estimated by the Kaplan-Meier function and contrasted with the Log-Rank test. The mean and median survival times and confidence intervals (95%) were also determined.

Overall survival was defined as the time elapsed from the vaccine starting day until death or the date of the last news. In addition, overall survival from first line therapy was estimated.

Progression-free survival was defined as the time interval between CIMAvax-EGF first dose to the disease progression.

## Results

Overall, 106 patients were included in the study. The median follow-up time was 14.2 months, 95% CI (11-15.8 months). Table 1 shows patients' characteristics as well as first line treatment, in addition to the response to the referred front line.

The predominant groups were patients with 65 years or older (52.8%), male-sex (58.5%), ECOG-1 (77.4%), adenocarcinoma histology (62.3%) and stage IIIB NSCLC (58.4 %). The combination of chemotherapy and radiotherapy was the most commonly used first line treatment (58.4 %). The most prevalent treatment modality was platinum plus etoposide (78.5%). Overall, 56.6% had a partial response to first-line treatment.

Median overall survival was estimated from the first line therapy and from the CIMAvax-EGF starting day. The median overall survival from the chemotherapy or chemo-radiotherapy initiation was 22.46 months, 95% CI (19.92-25.0) (graphic not shown) and the survival rates at 6, 12, and 24 months were 97.7 %, 82.7 % and 45.5 %, respectively.

**Figure 1:**
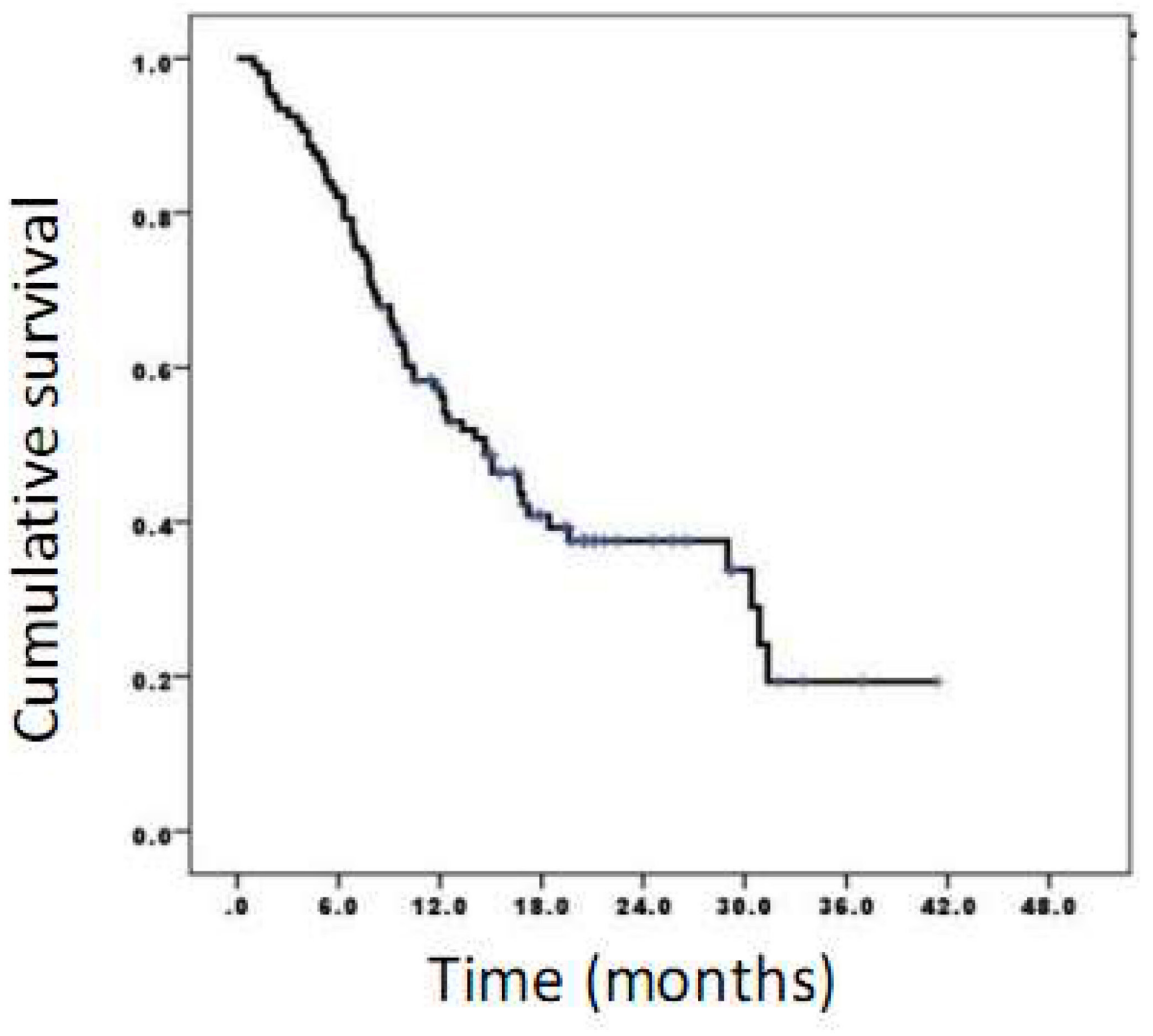
Survival time of patients vaccinated with CIMAvax-EGF. The median overall survival was 14.6 months while survival rates at 6, 12, and 24 months were 82.1%, 57.2%, and 37.6%, respectively.

The time lag between chemotherapy completion and CIMAvax-EGF ranged from 8 to 12 weeks. In addition, survival time from the first dose of CIMAvax-EGF was estimated. The median overall survival was 14.6 months, 95% CI (10.6-18.8) (Figure 1).

**Figure 2:**
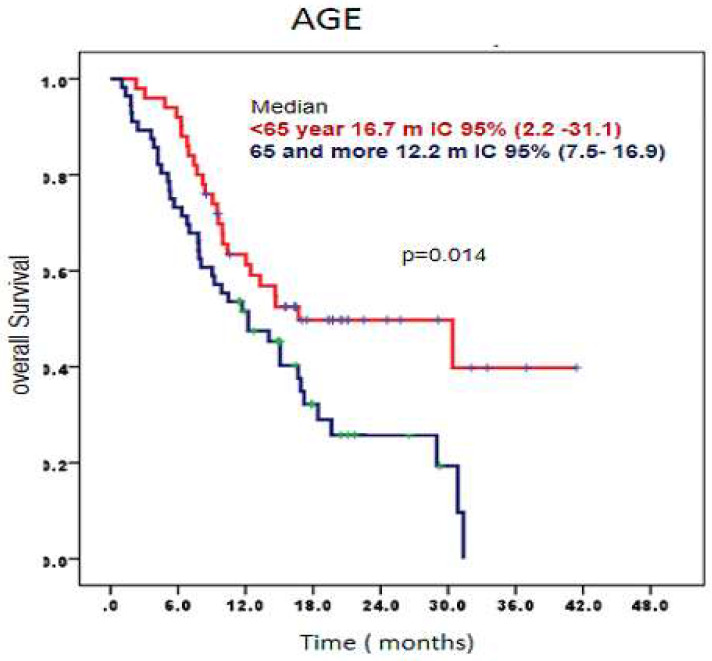
Survival time according age of patients treated with CIMAvax-EGF. Patients younger than 65 had a significantly larger overall survival.

When analyzing overall survival according age, patients younger than 65 years had a median overall survival of 16.7 months, 95% CI (2.2-31.1) compared to those older than 65 years who had a median of overall survival 12.2 months, 95% CI (7.5-16.9) (p= 0.014) (Figure 2).

**Figure 3:**
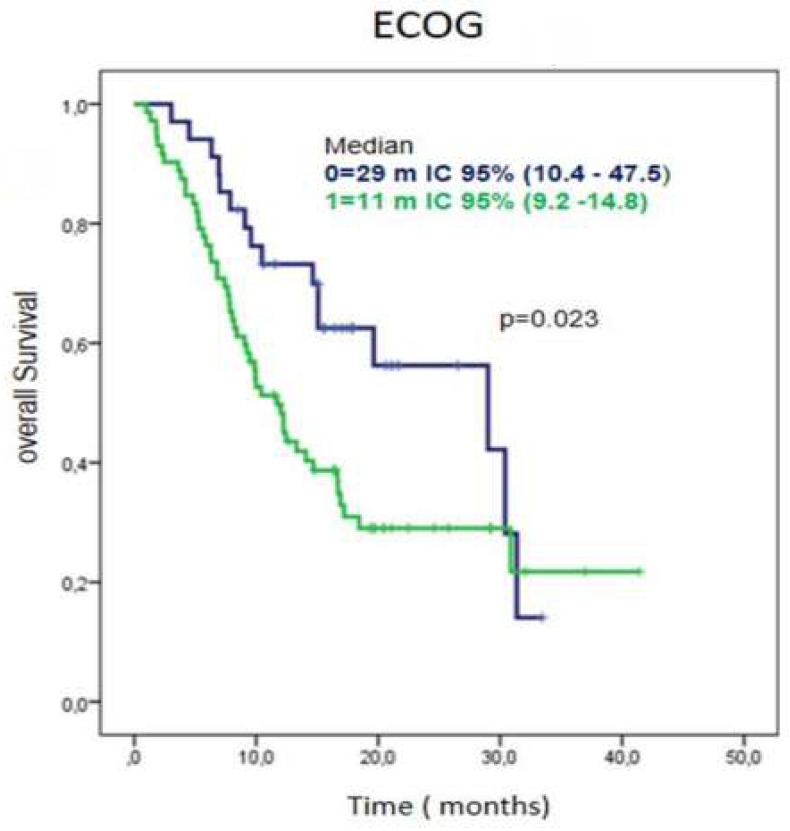
Survival time according ECOG in patients treated with CIMAvax-EGF. Patients with ECOG 0 had a significantly larger overall survival.

Regarding performance status, patients with ECOG 0 had a median overall survival of 29 months, 95% CI (10.4-47.5) while those with an ECOG-1 at diagnose, had a median overall survival of 11 months (95% CI 9.2-14.8) (Figure 3).

**Figure 4:**
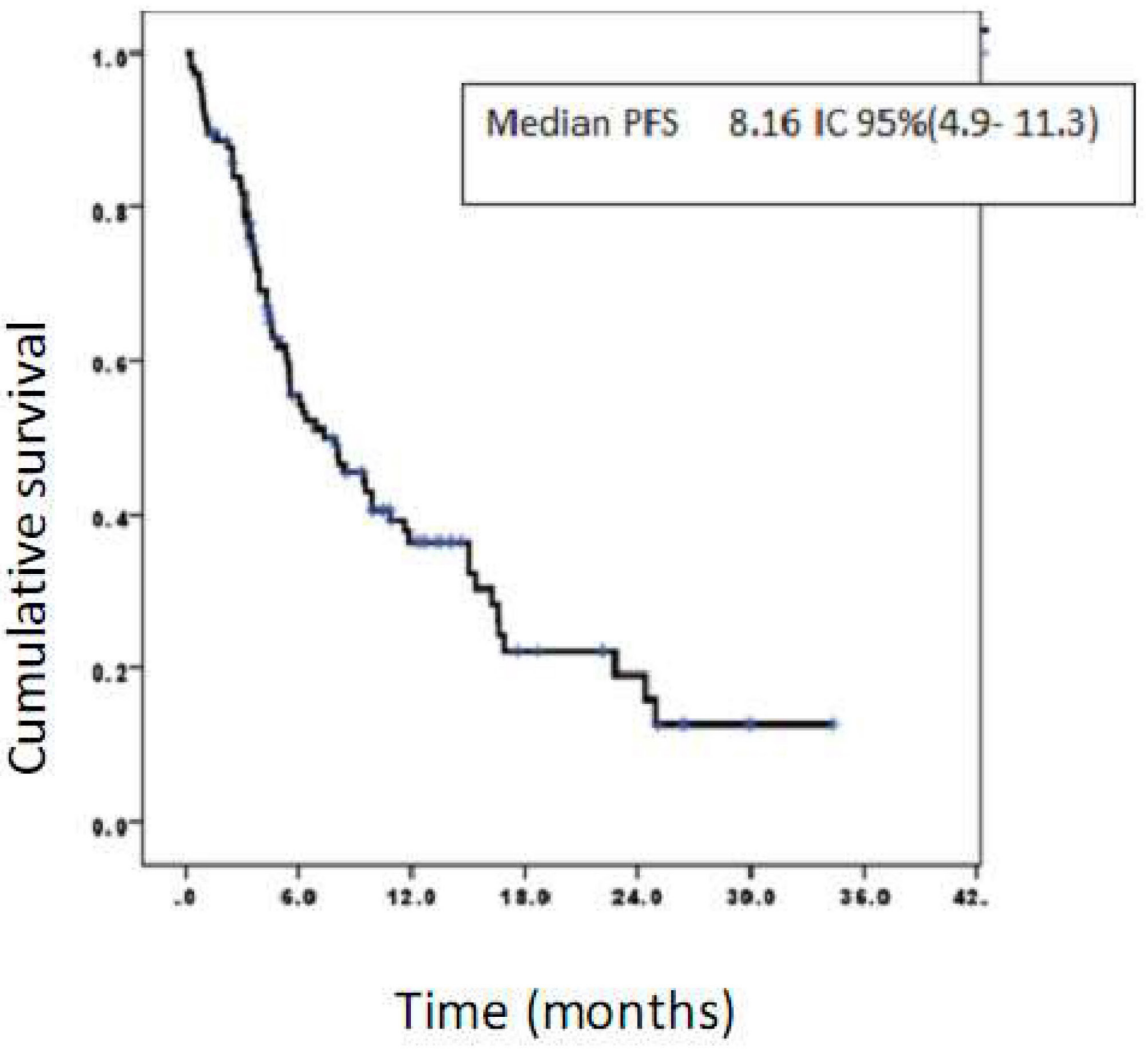
Progression-free survival of patients treated with CIMAvax-EGF. Progression-free survival rates at 6, 12, and 24 months were 55.4%, 36.4%, and 19.1%, respectively.

The median progression-free survival was 8.16 months, 95% CI (4.9-11.3) (Figure 4).

Globally, 36.8% and 19.8% of patients had objective response or disease stabilization at 6 and 12 months of treatment with CIMAvax-EGF, respectively (Table 2).

Treatment was safe as no serious adverse events were reported. The most frequent adverse events were grade 1 injection site pain (27.2 %) and local erythema (10.9 %). The most frequent grade 2 adverse event was vomiting (1.8 %) (Table 3).

## Discussion

The objective of this study was to characterize the safety and efficacy of an EGF depleting immunotherapy (CIMAvax-EGF) as maintenance therapy in the real-world setting. When considering the most relevant demographic and tumor variables, patients were similar to the ones included in the phase III trial, except for the histology. In our scenario, the predominant histology was adenocarcinoma, while Rodríguez et al. recruited mostly patients with squamous cell carcinomas 14. Other CIMAvax-EGF trials conducted by Ramos et al. 13 and Saavedra et al. 17 enrolled mostly adenocarcinomas, like our case.

Median overall survival (14.6 months) was larger than the one reported by Rodriguez and cols, in the multicentric phase III trial (10.8 months) 14. Patients younger than 65 had better survival. It is well validated that older subjects had a decreased ability to respond to vaccination and to fight infections. These patient population also have a constitutive low-grade inflammation 15. Elderly patients are more prone to an uncontrolled activation of innate immune response that leads to cytokine release syndrome and tissue damage 16.

In the phase II study, vaccinated patients with a good antibody response had a median overall survival of 11.7 months, significantly higher than the control group treated with best supportive care (5.33 months) 12. In the referred study, the 6- and 12-months survival rates were 82.1% and 57.2% 12.

Other maintenance therapy studies including pemetrexed and bevacizumab reported similar median survival times. Median survival time (MST) after pemetrexed was 15 months 18-20 while after bevacizumab, MST was 14.4 months 20 21. In the KEYNOTE 024 clinical trial that evaluated the impact of using pembrolizumab in patients with PD-L1 expression larger than 50 %, the median overall survival was 30 months 22. KEYNOTE 024 is not strictly comparable with our study, when considering the selection bias.

In our study, the median progression-free survival (PFS) was 8.16 months, higher than the PFS reported for pemetrexed (4-5 months), bevacizumab or gemcitabine 18-21, 6, 23. For bevacizumab as maintenance therapy, the median PFS was 4 months 6. Brodowicz et al. reported that gemcitabine used as continuation maintenance, had a PFS equivalent to 3.8 months 23. However, in the KEYNOTE 024 study, patients with high PD-L1 expression treated with pembrolizumab had a PFS of 10.3 months 22. In a different study including patients with PD-L1 of at least 1 % (KEYNOTE 189) the median PFS after using pembrolizumab as maintenance therapy was 8.8 months, similar to the patients treated with CIMAvax-EGF 5.

CIMAvax-EGF showed a disease control rate at 6 and 12 months of 36.8% and 19.8%. Ramalingam et al. found that the response rates after pemetrexed at the same time intervals were 18.5% and 12.5%, lower than the results seen in our study 20. Recently, the Impower 150 study comparing chemotherapy plus atezolizumab/bevacizumab (ABCP) followed by maintenance with atezolizumab and bevacizumab (BCP) or atezolizumab (ACP) alone also achieved good results. In arm A where atezolizumab was used alone, the objective response rate was 40.6% while in arm B, which uses the combination atezolizumab and bevacizumab, the response rate was 56.4 % 24. Improved progression-free survival with ABCP versus BCP or ACP was also observed. Median progression-free survival 8·4 months (ABCP) vs 6·8 months (BCP or ACP) 24. CIMAvax-EGF's PFS data in our study compares favorably with the above referred.

Characterizing the safety profile of the vaccine in the real-world setting is very important. Across the previous studies, including phase I, II and III clinical trials, no serious adverse events were reported. 12-14. In the phase II study, Neninger et al. found that the most common adverse events were grade 1-2 fever, headache, asthenia and injection site pain (13%). In this study, it was demonstrated that the vaccine was safe since no patients developed serious adverse events and the percentage of patients developing grade 1-2 adverse events was very small. Other maintenance trials with pemetrexed and bevacizumab, did find grade 3-4 adverse events 20. In the Impower 150 study, 64 % of the patients treated with the atezolizumab/bevacizumab plus chemotherapy, followed by atezolizumab and bevacizumab, had grade 3-4 adverse events 24.

One of the limitations of the current study is that serum EGF levels, as well as anti EGF antibodies, were not evaluated. Previous studies have shown that EGF is a predictive marker of CIMAvax-EGF efficacy 17. Better selection of the patient population would impact in a larger median PFS and overall survival.

## Conclusions

In conclusion, in our standard practice, CIMAvax-EGF as an EGF depleting immunotherapy used after front line therapy was safe and effective in patients with NSCLC.

## Figures and Tables

**Table 1:** Characteristics of the patients treated with CIMAvax-EGF. Most patients had ECOG-1, adenocarcinoma histology and partial or stable disease after completing front line therapy.

Patient Characteristics		No (106)	%
Age	65 years	50	47.2
	65 years and more	**56**	**52.8**
Gender	Male	**62**	**58.5**
	Female	44	48.5
ECOG	0	22	20.8
	1	**82**	**77.4**
	2	2	1.9
Histology	Adenocarcinoma	**66**	**62.3**
	Squamous cell	28	26.4
	Large cell carcinoma	12	11.3
Stage	IIIB	**63**	**59.4**
	IV	43	40.6
First line therapy	Chemotherapy Radiotherapy	**63**	**59.4**
	Chemotherapy	43	40.6
Chemotherapy	Cisplatin/etoposide	**83**	**78.3**
	Carboplatin/paclitaxel	13	12.2
	Carboplatin / gemcitabine	10	9.4
Response to first-line treatment	Complete response	8	7.5
	Partial response	**60**	**56.6**
	Stable disease	38	35.8

**Table 2:** Evaluation of the response at 6 and 12 months in patients treated with CIMAvax-EGF. Overall, 36.5 % and 19.8 % of all patients, maintained disease control after 6 or 12 months of vaccination, respectively.

	6 months		12 months	
Response	No	%	No	%
Complete response	3	2.8	3	2.8
Partial Response	10	9.4	4	3.7
Stable Disease	26	24.5	14	13.2
Progressive Disease	67	63.2	85	80.1
Global	106	100	106	100

**Table 3:** CIMAvax-EGF related adverse events. Most prevalent adverse events were injection site reactions including pain, erythema and induration*.*

Adverse Event	Grade 1		Grade 2	
	No	%	No	%
Pain in the site of injection	33	27.3	-	-
Local erythema	12	10.9	-	-
Nausea	5	4.5	-	-
Induration at the injection site	8	7.3	-	-
Dizziness	5	4.5	-	-
Vomiting	1	0.9	2	1.8
Fever	2	1.8	-	-
Others	6	5.5	-	-
All	72	67.9	2	1.8
